# The 675‐nm wavelength for treating facial melasma

**DOI:** 10.1111/srt.13434

**Published:** 2023-08-06

**Authors:** Luigi Coricciati, Maddalena Gabellone, Pantalea Delle Donne, Beatrice Marina Pennati, Tiziano Zingoni

**Affiliations:** ^1^ Coricciati Medical Group Martano Italy; ^2^ El.En. Group Calenzano Italy

**Keywords:** 675‐nm laser, facial melasma, pigmentary lesions, vascular lesions

## Abstract

**Background:**

Melasma is an acquired skin problem. It characterises sun‐exposed areas, particularly on the face, with irregular borders and bilateral distribution. With this study, we want to strengthen the scientific literature regarding the use of a 675‐nm laser device for the treatment of women and men with facial melasma pigmentary and vascular symptoms.

**Materials and methods:**

Eighteen patients were treated for facial melasma. A total of three sessions at 30‐day intervals were performed with a 675‐nm laser device. A five‐point Global Aesthetic Improvement Scale was used to separately assess the improvement of the patient's skin 3 months after the last treatment (T1) compared to baseline (T0). The pain during treatment was measured using a Visual Analog Scale of 10 points. The non‐ablative laser system used emits red light with a wavelength of 675 nm through a 13 × 13 mm scanning system.

**Results:**

At T_1_, a consistent improvement in the pigmentary and vascular components was visible. This is always combined with a considerable reduction in vascular expression.

**Conclusion:**

Our research shows that individuals with Fitzpatrick phototypes II to III can treat facial melasma with the 675‐nm laser source system without risk. Due to its interaction with melanin, collagen and haemoglobin chromophores, as well as its excellent capacity to penetrate tissues with less heating, this system is promising in the treatment of pigmentary and vascular illnesses such as melasma. The great success of the technology we used came from the reduced levels of inflammation produced after the treatments and the low energy level implied.

## 1 INTRODUCTION

Melasma is an acquired skin problem that can affect up to 30% of the population in certain world areas such as Southeast Asia or Latin America.^1^ Indeed, it is frequent to find hyperpigmented macules on the face of people with darker Fitzpatrick phototypes IV–VI, especially in women (90% of patients). It characterises sun‐exposed areas, particularly on the face, with irregular borders and bilateral distribution. The centrofacial, malar and mandibular forms of melasma are the three most common.^2^ Melasma is categorised as epidermal, dermal, or mixed form based on the location of the pigment by Wood's lamp examination.^3^

Even if the ultraviolet light exposition and hormonal influences seem to be the most influencing factors in the appearance of symptoms, the aetiology is probably multifactorial and not too clear.^4^ Indeed, when compared to healthy volunteers, melasma patients have greater melanogenesis and higher markers of oxidative stress, suggesting that UV light both initiates and contributes to the condition.^5^ Moreover, the idea that melasma is caused by a genetic predisposition is supported by the knowledge that family history is a significant risk factor for getting the condition. Fitzpatrick phototype II and III patients, for instance, are less likely to have a favourable family background than patients with darker skin types (IV–VI).^6^ The increased prevalence of melasma with pregnancy, oral contraceptive use and other hormonal therapies, as well as the greater expression of the progesterone receptor in the epidermis of the affected skin, all point to the pathogenesis of the disease that it is significantly influenced by hormones. An example is given by the high expression of estrogenic receptor proteins in the skin and around the blood vessels.^7^, ^8^ In addition, the release of the stem cell factor (SCF), a ligand for the tyrosine kinase receptor c‐kit, is one of the key mechanisms of both UV and visible light‐induced pigmentation, and they have ultimately effects on melanocyte proliferation. It is typical to also have increased mRNA levels of melanogenesis‐associated genes and increased SCF expression in melasma‐affected regions.^9^ Increased expression of genes linked to Wnt signalling is also noted. Indeed, the Wnt pathway has been connected to the development of melanocyte stem cells as well as the Vascular endothelial growth factor (VEGF), produced by keratinocytes in response to UV damage.

Kang et al. found that 279 genes were differently expressed in lesional skin. Specifically, they identified four upregulated melanogenesis‐associated genes in the subset of Wnt pathway modulators. Indeed, this pathway is crucial in the development of melanocytes in the epidermis.^9^

Melasma frequently has detrimental psychological effects on patients and reduces their quality of life. Patients frequently feel humiliated, low on themselves, bored, unsatisfied and lacking social drive.^10^

For these reasons, nowadays, many strategies to solve the photodamage, inflammation, vascularity and pigmentation related to melasma have been used. Topical (including photoprotection) and oral medications, chemical peels and lasers or a combination of these treatments, are the most common solutions to the problem. It is important to underline that none of these treatments can fully solve facial melasma symptoms.

Specifically, laser therapy has emerged as a viable option for patients with refractory cases of melasma compared to first‐line therapies. The efficacies and side effects of a wide variety of different laser therapies have been examined in numerous clinical trials up to this point. Intense pulsed light (IPL), Q‐switched lasers, picosecond lasers, non‐ablative fractionated resurfacing lasers and ablative fractionated resurfacing lasers are the five main types of lasers and light therapy.^11^

They are proven to be very effective, but downtime is often long, and people cannot immediately get back to everyday life. According to the existing literature, the specific 675‐nm wavelength has previously been shown to be ideal for treating acne scars, facial ageing and skin resurfacing.^12^, ^13^ Indeed, its emission in the red spectrum has a high affinity with collagen fibres and melanin chromophore, and a minimal interaction with the vascular chromophore.^14^ In this manner, by minimising the interaction with other chromophores, the laser beam immediately transfers heat to the collagen fibres, resulting in shrinkage and denaturation with subsequent neocollagenogenesis that has been histologically proven.^15^

Nevertheless, there are still not many research studies focused on the effect of 675‐nm wavelength and the improvement of the skin vascular component. For example, Nisticò et al. showed the effectiveness of a 675‐nm laser device on melasma‐affected women with Fitzpatrick skin types I–III.^16^, ^17^

Capillaries are the smallest vessels in the human body, and they have a simple and unique structure. They consist of two layers only, an inner monolayer of endothelial cells and an outer basal membrane (BM or basal lamina). Specifically, the BM is a 50–100‐nm layer of specialised extracellular matrix (ECM), mostly composed of collagen (type IV) and laminin.

With this study, we want to strengthen the scientific literature regarding the use of a 675‐nm laser device for the treatment of women and men with facial melasma pigmentary and vascular symptoms. We think that due to the laser's high affinity with collagen and melanin, and the typical anatomical capillary structure, it can be very effective for benign pigmented lesions, reducing the risk of side effects and simplifying post‐treatment management.

## 2 MATERIAL AND METHODS

### 2.1 Study population

In this study, 18 patients (16 females and 2 men), with a mean age of 44.78 ± 5.62, were treated for pigmented and vascular symptoms of facial melasma. They were 83% Fitzpatrick phototype II and 27% Fitzpatrick phototype III (see Table 1 for details). The VISIA imaging system (Canfield) was used to acquire pictures of the facial area of every patient. For each of them, three pictures were taken to evaluate skin appearance in visible light, and a specific software was implied to underline the pigmentary/melanin vascular components improvement.

**TABLE 1:** Population general characteristics.

No.	Age	Sex	Fitzpatrick phototype	Area	Treatment	No. of treatments	Pain(Vas 0–10)	Side effects
1	27	F	2	Face	Melasma	3	2	Daily erythema
2	43	F	2	Face	Melasma	3	3	Daily erythema
3	40	F	2	Face	Melasma	3	2	Daily erythema
4	45	F	3	Face	Melasma	3	3	Daily erythema
5	42	M	2	Face	Melasma	3	2	Daily erythema
6	46	F	3	Face	Melasma	3	2	Daily erythema
7	47	F	2	Face	Melasma	3	2	Daily erythema
8	47	F	2	Face	Melasma	3	2	Daily erythema
9	45	F	2	Face	Melasma	3	2	Daily erythema
10	41	F	3	Face	Melasma	3	1	Daily erythema
11	46	M	2	Face	Melasma	3	2	Daily erythema
12	49	F	3	Face	Melasma	3	1	Daily erythema
13	49	F	2	Face	Melasma	3	1	Daily erythema
14	40	F	2	Face	Melasma	3	1	Daily erythema
15	49	F	2	Face	Melasma	3	1	Daily erythema
16	50	F	2	Face	Melasma	3	2	Daily erythema
17	51	F	3	Face	Melasma	3	1	Daily erythema
18	49	F	2	Face	Melasma	3	4	Daily erythema

A total of three sessions at 30‐day intervals were performed with a 675‐nm laser device (RedTouch, Deka M.E.L.A.). A five‐point Global Aesthetic Improvement Scale (GAIS) (None: 0; mild/25%: 1; moderate/50%: 2; good/75%: 3; excellent/100%: 4) was used to separately assess the improvement of the patient's skin 3 months after the last treatment (T1) compared to baseline (T0), considering the picture without filters, and with the brown (pigmentary) and red (vascular) filters as performed by Piccolo et al.^18^ The pain during treatment was measured using a Visual Analog Scale of 10 points, with 0 (‘no pain’) and 10 (‘pain as bad as it could possibly be’).

All subjects gave their informed consent before the study began.

### 2.2 Laser device

**FIGURE 1:**
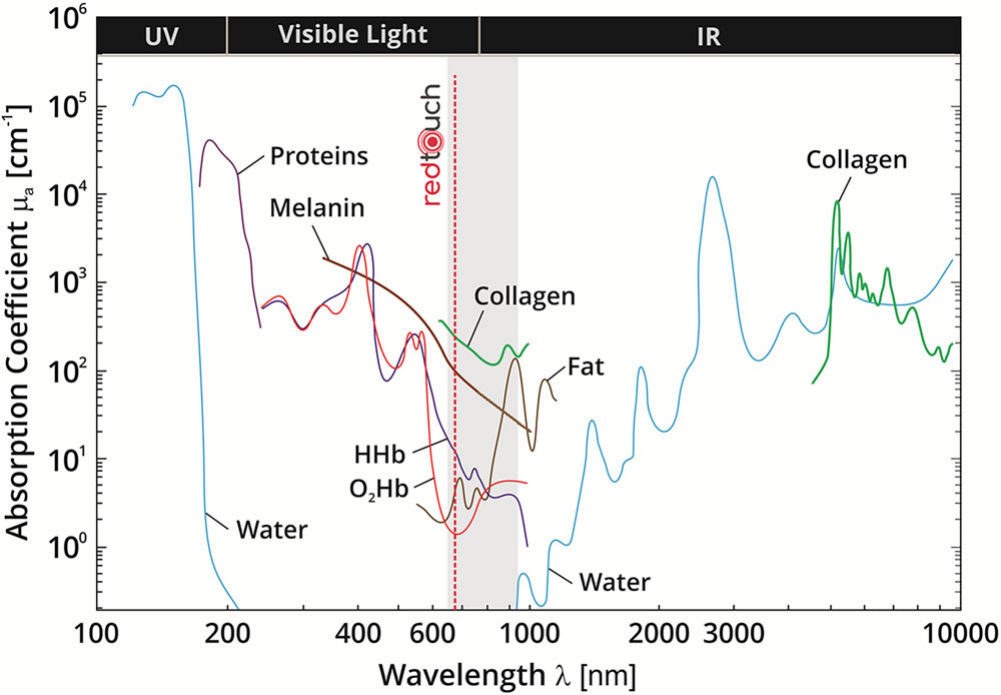
Absorption spectra (logarithm base) for different chromophores present in human tissue. The spectra of oxyhaemoglobin (O_2_Hb), human haemoglobin (HHb), proteins, water, collagen, fat and cytochrome oxidase (CtOx) are shown. It is possible to appreciate the range of action of the RedTouch device. It shows affinity with collagen, melanin and haemoglobin as well. Picture modified from Scholkmann et al. (2014).^19^

According to preclinical tests, the non‐ablative laser system (Redtouch, Deka M.E.L.A.) used emits red light with a wavelength of 675 nm through a 13 × 13 mm scanning system that can target collagen and melanin with precision (see Figure 1).

It can be optimised with a contact and temperature sensor included in the handpiece. This can produce fractional micro‐zones (DOT) measuring 0.7‐mm wide of either selective thermal or sub‐ablative damage on the skin. Through the Power and DOT pulse duration (Dwell time) parameters, each DOT is exposed to radiation from the laser source. The scanning technology partially covers the treatment area uniformly by introducing a spacing (Spacing) between each DOT. Each emission may penetrate a thermal column more than 1‐mm deep. Various factors can be used to control the delivered energy (power, pulse duration and distance between microthermal zones). Indeed, the device is equipped with a skin cooling system to avoid heat‐induced damage to the epidermis and reduce downtime.

### 2.3 Laser treatment protocol

The patient's face was washed with gentle soap and water prior to treatment. The endpoint expected was a slight erythema with accompanying oedema. Each patient underwent an energy therapy assessment on a particular area 'test' based on their skin type and level of tolerance. The endpoint was identified as mild erythema. The handpiece was gently passed over the skin's surface in locations that were near to one another, without overlapping, and without treating any untreated areas.

The following parameters were used: power 10 W, pulse duration 150 ms, spacing 1500 mm, and stack 1. A transparent conductive gel was used to administer the treatment. Sessions were held at intervals of 30 days. Treatment was carried out by passing the handpiece over the interested areas while applying light pressure, and no overlapping. Right after the treatment, cold water‐soaked gauzes were used to cool down the skin.

### 2.4 Post‐treatment care

A hydrating, soothing and protective water‐based solution (URIAGE—Eau Thermal) was daily applied to the treated area to help rebuild the skin barrier and maintain an optimum moisturisation level.

Side effects such as blistering, scarring, burns, hypopigmentation or hyperpigmentation were monitored during and right after every treatment.

## 3 RESULTS

None of the above‐mentioned side effects was noted during or right after any treatment. Just a mild erythema lasting for a few hours after treatment was present.

**FIGURE 2:**
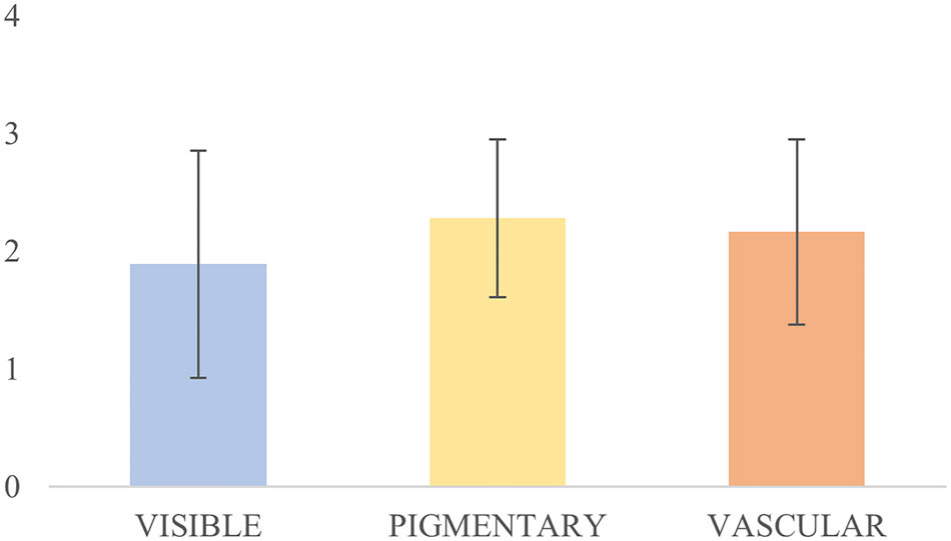
A five‐point Global Aesthetic Improvement Scale (GAIS) (None: 0, mild/25%: 1, moderate/50%: 2, good/75%: 3, excellent/100%: 4) was used to evaluate the patients’ skin improvement before and 3 months after the last treatment (T_1_) with RedTouch. The clinical pictures in visible light (blue column), with the pigmentary brown filter (yellow column) and with the vascular red filter (orange column) were considered.

For all patients, it was possible to evaluate skin improvement before and after the last treatment (3‐month follow‐up) with RedTouch. In general, at T_1_ a consistent improvement in the pigmentary and vascular components was visible. Indeed, the GAIS for visible, pigmentary, and vascular values were 1.89 (±0.96), 2.28 (±0.67) and 2.17 (±0.79), respectively (see Figure 2).

In general, there was apparently more consistent improvement in the pigmentary and vascular components compared to the visible result (moderate to good). This is due to the graphical elaboration the pictures underwent. So, Figure 2 must be interpreted as separating the filtered pictures from the unfiltered ones.

**FIGURE 3:**
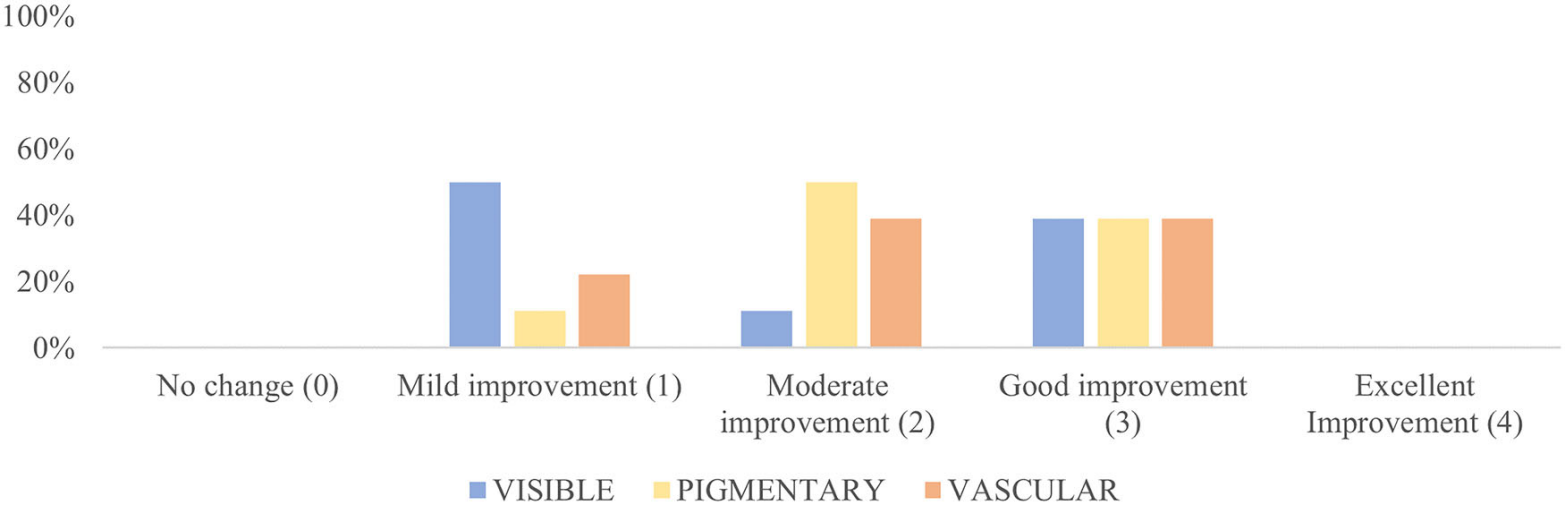
Frequency histograms. Data regarding the pictures analysed in visible light (blue column), with the pigmentary brown filter (yellow column) and with the vascular red filter (orange column) were considered.

None of the patients reported no results (see Figure 3).

**FIGURE 4:**
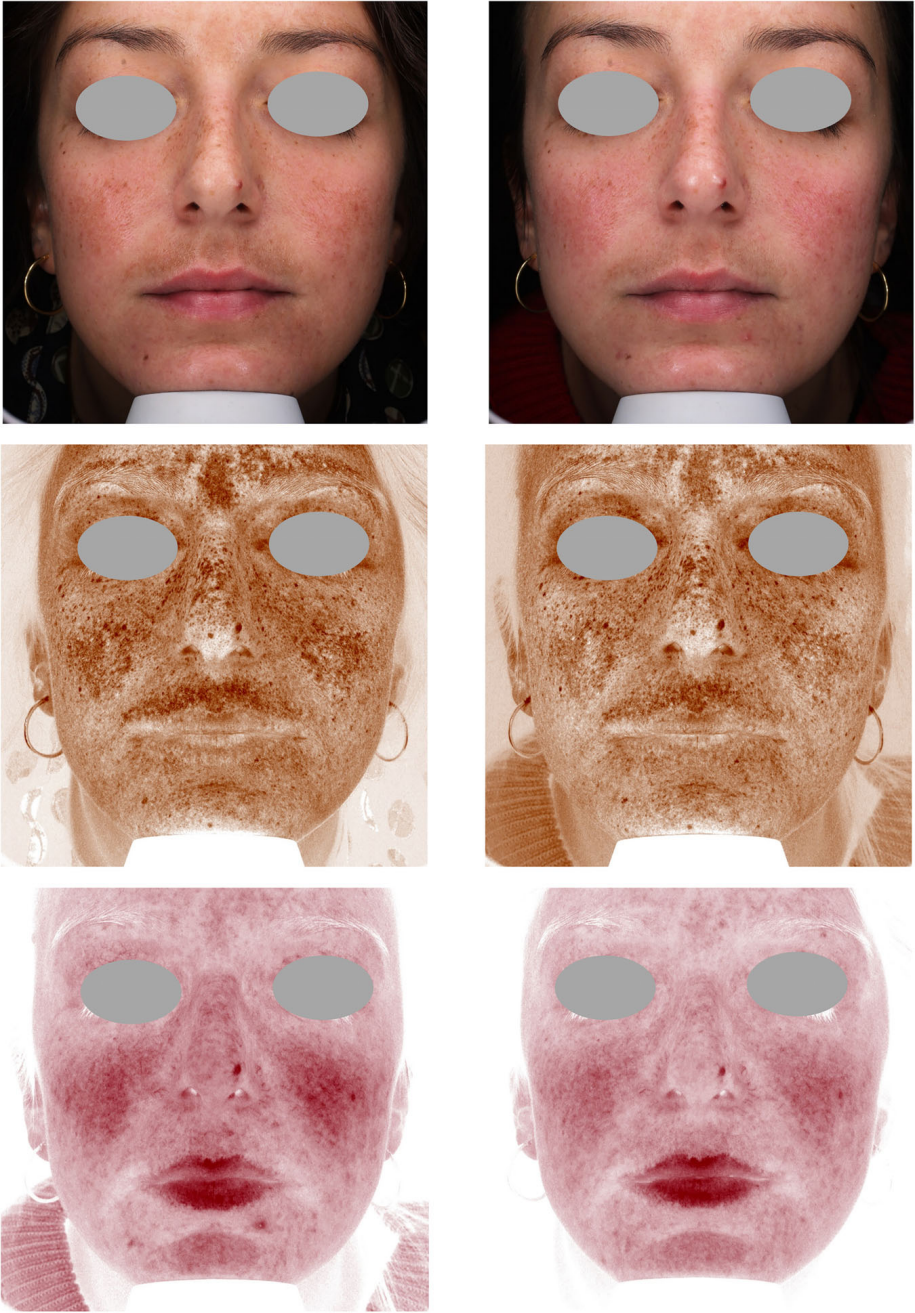
The VISIA imaging system (Canfield) was used to acquire pictures of the facial area of female (4–7) and male (8) patients. The improvement of the patient's skin after the last treatment (T_1_, after 3 months) compared to baseline (T_0_) was considered. Pictures without filters (visible), with brown (pigmentary) and red (vascular) filters, are displayed.

**FIGURE 5:**
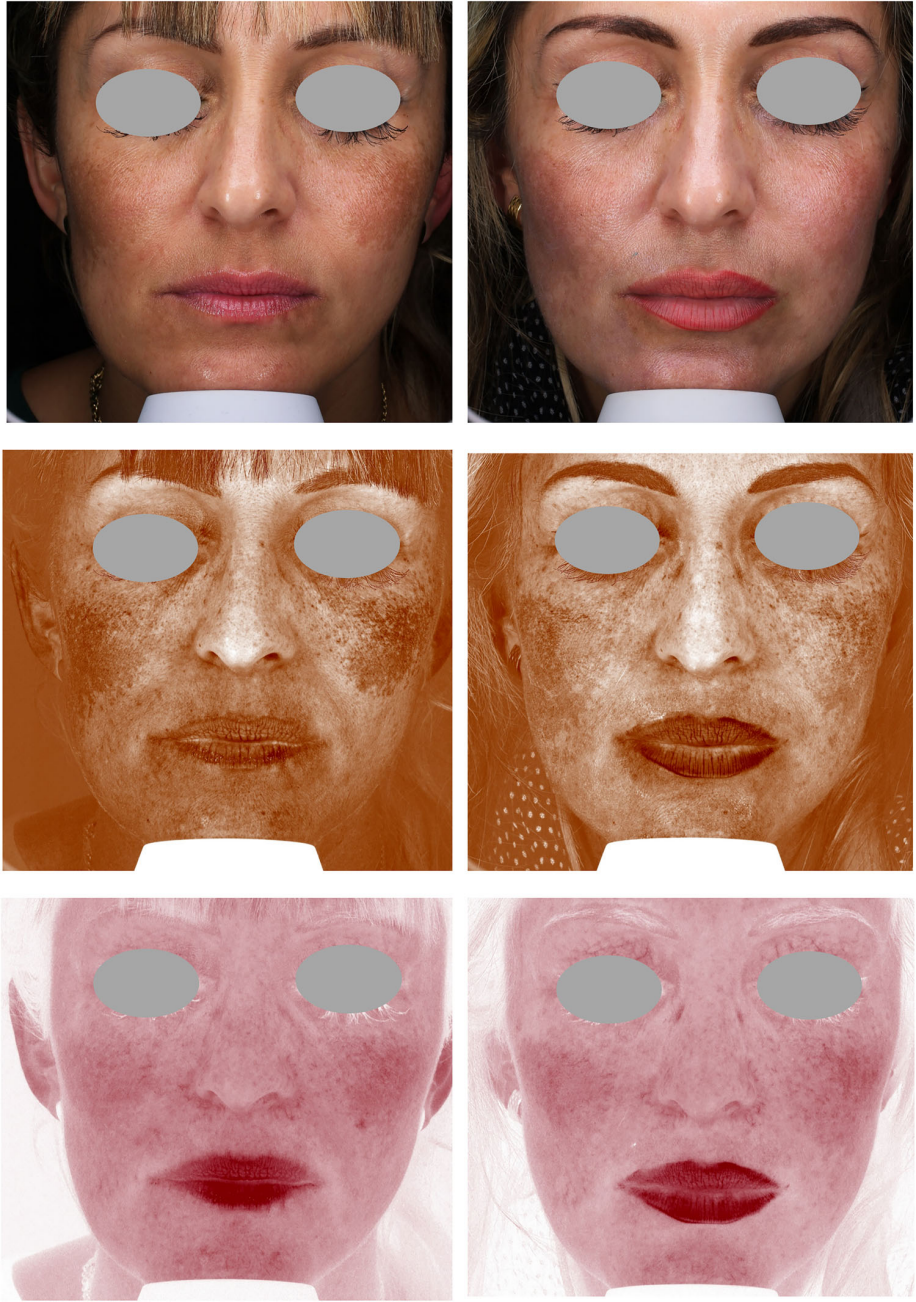
The VISIA imaging system (Canfield) was used to acquire pictures of the facial area of female (4–7) and male (8) patients. The improvement of the patient's skin after the last treatment (T_1_, after 3 months) compared to baseline (T_0_) was considered. Pictures without filters (visible), with brown (pigmentary) and red (vascular) filters, are displayed.

**FIGURE 6:**
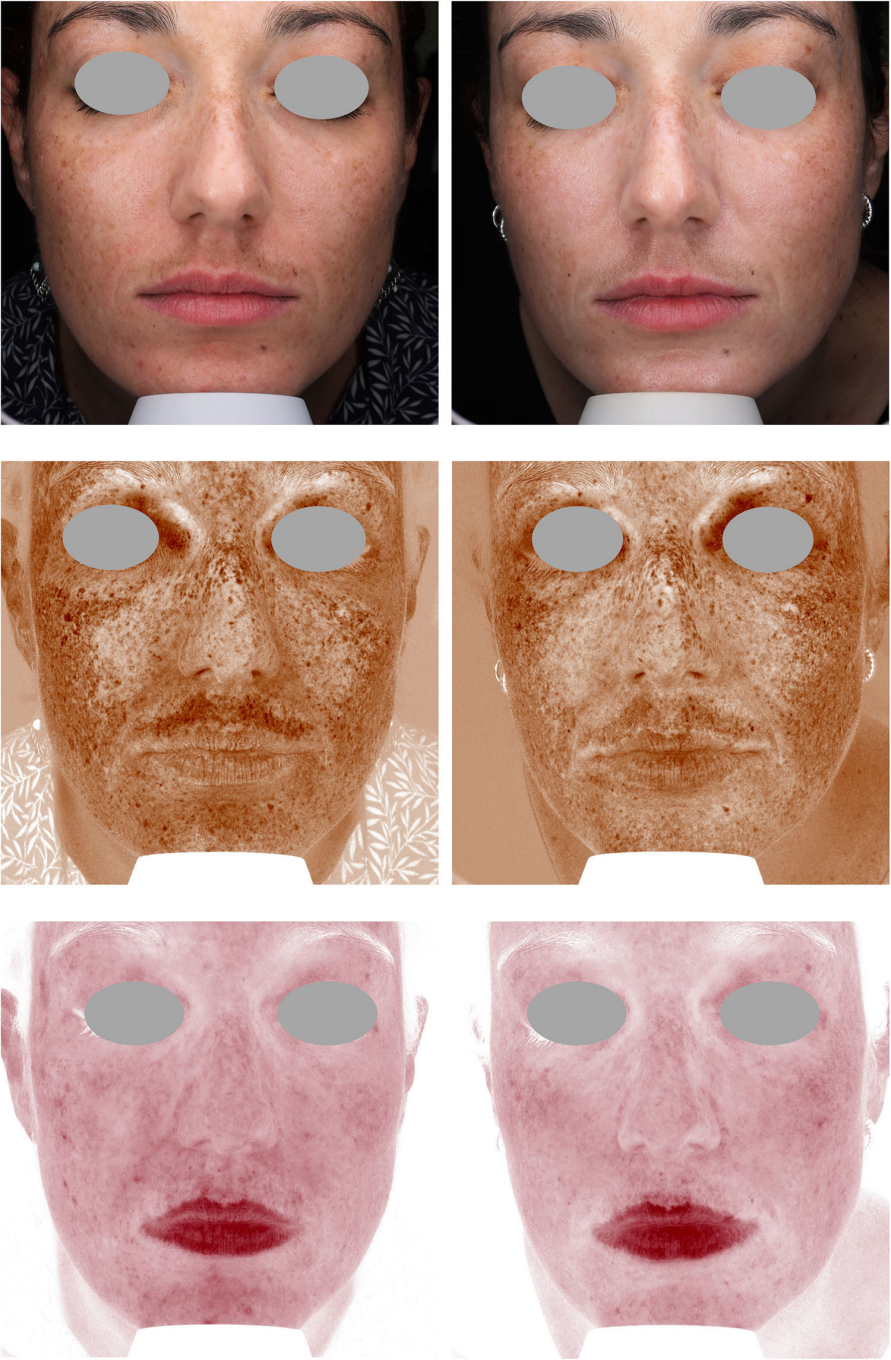
The VISIA imaging system (Canfield) was used to acquire pictures of the facial area of female (4–7) and male (8) patients. The improvement of the patient's skin after the last treatment (T_1_, after 3 months) compared to baseline (T_0_) was considered. Pictures without filters (visible), with brown (pigmentary) and red (vascular) filters, are displayed.

**FIGURE 7:**
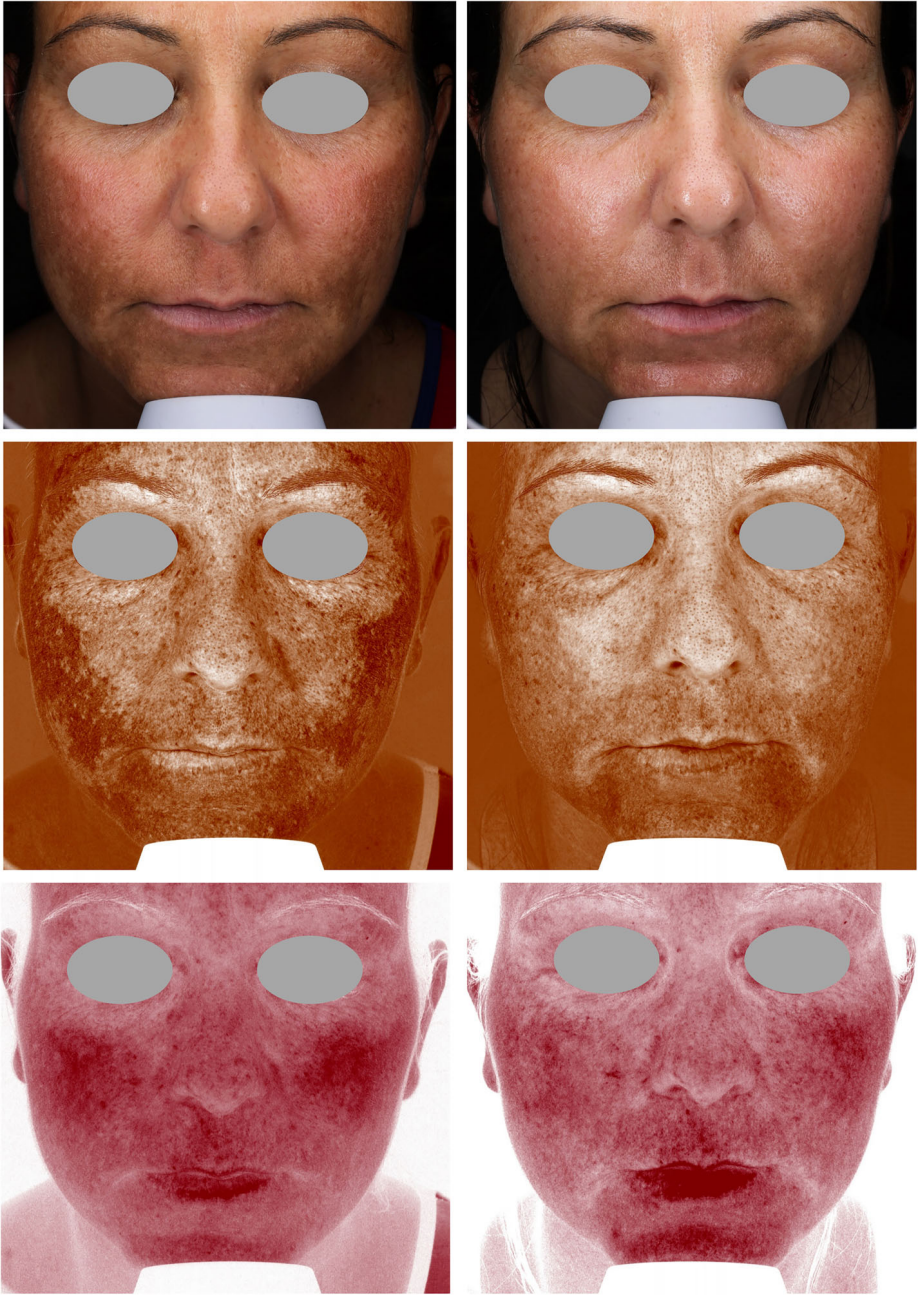
The VISIA imaging system (Canfield) was used to acquire pictures of the facial area of female (4–7) and male (8) patients. The improvement of the patient's skin after the last treatment (T_1_, after 3 months) compared to baseline (T_0_) was considered. Pictures without filters (visible), with brown (pigmentary) and red (vascular) filters, are displayed.

**FIGURE 8:**
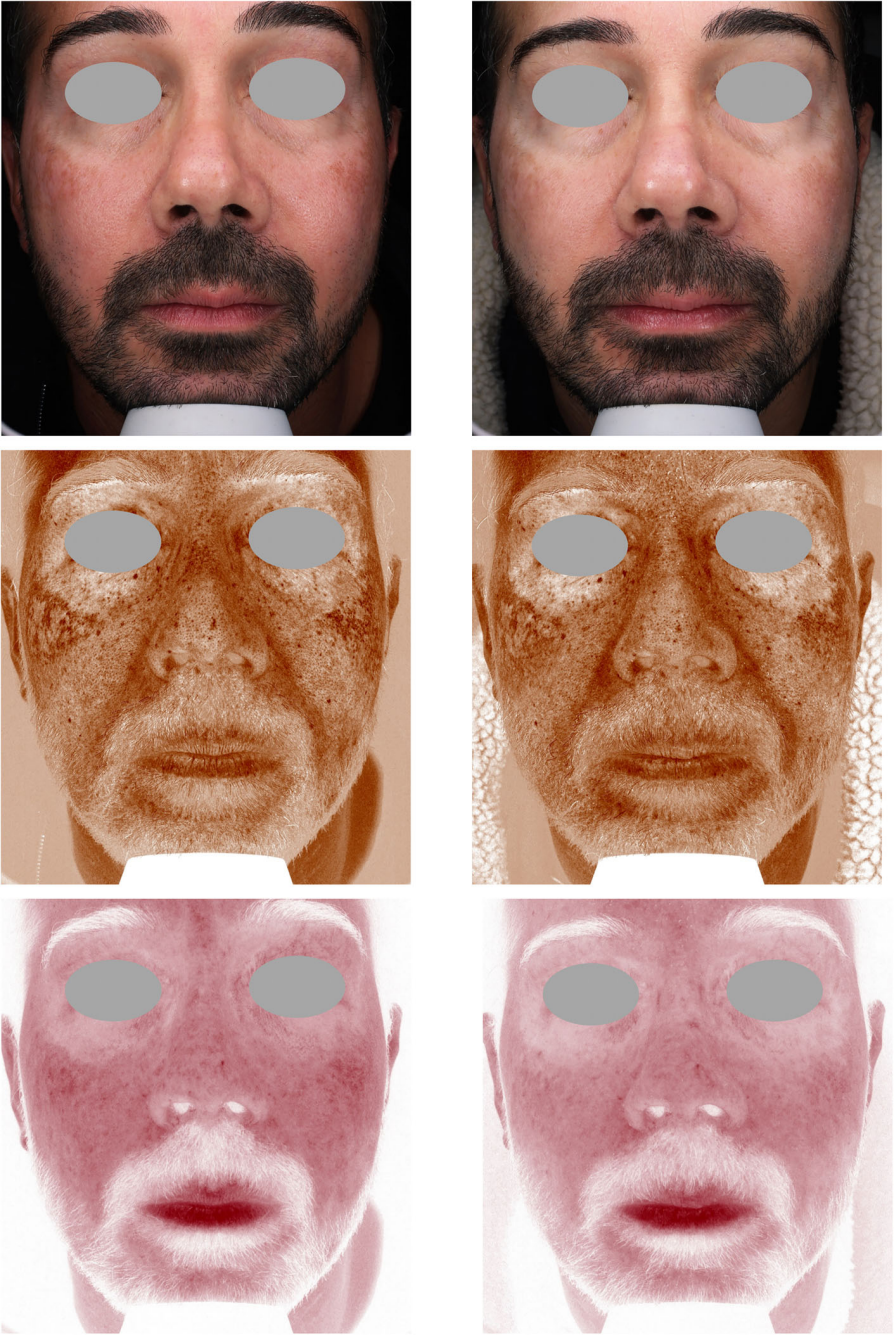
The VISIA imaging system (Canfield) was used to acquire pictures of the facial area of female (4–7) and male (8) patients. The improvement of the patient's skin after the last treatment (T_1_, after 3 months) compared to baseline (T_0_) was considered. Pictures without filters (visible), with brown (pigmentary) and red (vascular) filters, are displayed.

Figures 4, 5, 6, 7, 8 show some exemplificative before‐and‐after female and male cases. It is evident that a consistent reduction in the pigmentary component is visible in all subjects. This is always combined with a considerable reduction in vascular expression.

All patients tolerated treatment well (pain score: 1.89 ± 0.83). Most patients reported pain associated with the procedure as mild.

## 4 DISCUSSION

Combining various therapeutic approaches is the best therapeutic outcome for treating melasma. These include depigmenting products, stringent photoprotection, chemical (i.e., tranexamic acid) and physical treatments.^20^ Depigmenting agents can generally affect the skin on a variety of levels, including tyrosinase activity and transcription, melanosome transfer, melanin and melanosome degradation, and accelerated turnover of pigmented keratinocytes.

In this scenario, several research studies have been conducted on the use of laser technology in the treatment of melasma. The first methods relied on intense pulsed light and ablative lasers^21^, ^22^, ^23^ (such as CO_2_ and erbium), but both carried a significant risk of consequences, including post‐inflammatory hyperpigmentation that persists.^24^ Additionally, it has been shown that Q‐switched lasers reduce symptoms while minimising the risk of complications and adverse effects for patients. The Nd:YAG proved to be the best performer Q‐switched laser to treat melasma, because its wavelength of 1064 nm allows for reaching deeper layers with minimal damage to the epidermis, inflammatory stimulus, and risk of post‐inflammatory hyperpigmentation.^25^, ^26^, ^27^

Even intense pulsed light (IPL) has been demonstrated to be effective for treating melasma, but hyperpigmentation is a consistently unwanted problem over time. Moreover, relapses are still inescapable, so therapy combinations like IPL and Nd:YAG laser 1064‐nm low frequency typically are one of the solutions that yield quicker and more successful results.^27^

A significant global aesthetic improvement (GAIS score) in the pigmentary, vascular and visible components was observed at a 3‐month follow‐up. Nevertheless, none of the patients had no benefit or, on the contrary, an excellent improvement in the visible, pigmentary or vascular component. This confirms that until this moment, melasma treatment with the 675‐nm wavelength remains partial, even if at a 3‐month follow‐up a good outcome with no relapse was noted.

The great success of the technology we used comes from the reduced levels of inflammation produced after the treatments. This is due to specific technical characteristics of the system. First, the high selectivity for the melanin chromophore and the vascular vessel wall (as shown in Figure 1). Second, the possibility of reaching very low energy levels, even lower than those used for facial skin rejuvenation^13^ or treatment of acne scars.^12^ Indeed, compared to the research of Cannarozzo et al. (2021)^12^ and considering patients with the same phototype, the energy amount we used was one‐third of the one implied for improving facial acne scars.

We hypothesise that the vascular improvement effect reported after the treatment with the study device on the face area could be due to the presence of a collagen component in the capillary basal membrane. Indeed, even if the collagen type is different compared to bigger vessels (type IV vs. type I/III) it is still a target of the device wavelength. For this reason, the capillary collagen, when hit by the laser, undergoes a shrinkage effect, reducing the vessel lumen and so the blood flux. Moreover, Mathew‐Steiner et al. (2021)^28^ reported that types IV and XVIII of collagen show anti‐angiogenic properties,^29^, ^30^ inhibiting the proliferation and migration of endothelial cells and inducing endothelial cell apoptosis. Probably, these effects are influenced and amplified by the presence of collagen in the surrounding environment (connective tissue).

The procedure is simple, non‐invasive and has few adverse effects. When the treated areas' skin is properly cooled before applying the laser, the treatment is painless. Indeed, our patients well tolerated the treatment, reporting mild and bearable pain, resulting in a mean pain score of 1.89 ± 0.83 (range 0–10). Overall, these results are found both in men and women patients, regardless of the patient's skin phototype. Further studies are needed to understand the molecular basis behind the interaction of the 675‐nm wavelength and the vascular component, collagen especially.

### 4.1 Study limitations and future perspectives

Limitations of this work include the absence of a control group and long‐term follow‐ups, and it would be fascinating to enrich the research with a histological investigation.

## 5 CONCLUSION

Our research shows that individuals with Fitzpatrick phototypes II–III can treat facial melasma with the 675‐nm laser source system without risk. Due to its interaction with melanin, collagen and haemoglobin chromophores, as well as its excellent capacity to penetrate tissues with less heating, this system is promising in the treatment of pigmentary and vascular illnesses such as melasma. The great success of the technology we used came from the reduced levels of inflammation produced after the treatments and the low energy level implied. For these reasons, there were few side effects, excellent post‐treatment management, and no additional post‐inflammatory hyperpigmentation.

## CONFLICT OF INTEREST STATEMENT

Beatrice Marina Pennati and Tiziano Zingoni are employed at El.En. Group. The remaining authors declare that the research was conducted in the absence of any commercial or financial relationships that could be construed as a potential conflict of interest.

## ETHICS STATEMENT

The study was conducted in accordance with the Declaration of Helsinki. As the device has been an already CE‐marked device since 2019, ethical review and approval were waived for this study. Informed consent was obtained from all subjects involved in the study.

## Data Availability

The data that support the study findings are available on request from the corresponding author.
